# Was sollte der (Allgemein‑/Viszeral‑)Chirurg über Arbeitsmedizin wissen? Allgemeine Impfempfehlungen und postexpositionelle Prophylaxe von Hepatitis B, C und HIV

**DOI:** 10.1007/s00104-021-01502-w

**Published:** 2021-09-15

**Authors:** Beatrice Thielmann, Frank Meyer, Irina Böckelmann

**Affiliations:** 1 Bereich Arbeitsmedizin, Medizinische Fakultät, Otto-von-Guericke-Universität Magdeburg, Leipziger Straße 44, 39120 Magdeburg, Deutschland; 2 Universitätsklinik für Allgemein‑, Viszeral‑, Gefäß- und Transplantationschirurgie, Medizinische Fakultät, Otto-von-Guericke-Universität Magdeburg, Leipziger Straße 44, 39120 Magdeburg, Deutschland

**Keywords:** Schutzimpfung, Postexpositionsprophylaxe (PEP), Primärprophylaxe, Berufskrankheit, Prävention, Arbeitsunfall, Notfall, Infektiöser Indexpatient, TOP-Prinzip, Vaccination recommendation, Postexposure prophylaxis (PEP), Primary prophylaxis, Occupational disease, Prevention, Occupational accident, Emergency, Infectious index patient, TOP principle

## Abstract

In dieser Arbeit geht um die allgemeinen Impfempfehlungen und postexpositionelle Prophylaxe, die bei Schnitt- und Nadelstichverletzungen infrage kommen können. Schnitt- und Nadelstichverletzungen gehören zu häufigen Arbeitsunfällen bei chirurgisch bzw. operativ und interventionell tätigen Ärzten. Diese sind als Akut- bzw. Notfälle zu deuten, vor allem wenn Kontakt zu infektiösen Indexpatienten bestand bzw. die Infektionsgefährdung nicht auszuschließen ist. Folgend entstehen hohe volkswirtschaftliche Kosten, aber auch die individuelle Konfrontation mit einer durchaus nicht heilbaren Infektionskrankheit. Ziel dieser Übersichtsarbeit ist es, auf die allgemeinen und berufsbedingten Impfempfehlungen für Chirurgen hinzuweisen. Darüber hinaus werden Hintergründe und rechtliche Grundlagen dargestellt, denn auch für jeden Chirurgen besteht eine Hol- und Bringschuld im Rahmen effektiver Schutzmaßnahmen gegen Infektionskrankheiten durch Schnitt- und Nadelstichverletzungen. Ergänzend werden Primärprophylaxe, Impfempfehlungen und Postexpositionsprophylaxe nach stattgehabten Stich- oder Schnittverletzungen sowie das TOP-Prinzip aufgezeigt. Das TOP-Prinzip umfasst technische, organisatorische und personenbezogene Schutzmaßnahmen.

## Abstract (en)

This article deals with the general recommendations on vaccination and postexposure prophylaxis, which can be utilized for cuts and needlestick injuries. Cuts and needlestick injuries among surgeons are common occupational accidents. These should be interpreted as acute or emergency situations, especially if there was contact with infectious index patients or the danger of infection cannot be excluded. This results in high economic costs but also in the individual confrontation with an infectious disease that is definitely incurable. The aim of this review is to highlight the general and occupational vaccine recommendations for surgeons. In addition, background information and legal principles are presented. There is a duty to provide and to obtain information about effective protective measures against infectious diseases from cuts and needlestick injuries even for surgeons. In addition, primary prophylaxis, vaccination recommendations, and postexposure prophylaxis after cuts and needlestick injuries as well as the TOP principle are presented. The TOP principle comprises technical, organizational and person-related protective measures.

## Hintergrund

Schnitt- und Nadelstichverletzungen gehören zu häufigen Arbeitsunfällen bei chirurgisch bzw. operativ und interventionell tätigen Ärzten. Diese sind als Akut- bzw. Notfälle zu betrachten, vor allem wenn Kontakt zu infektiösen Indexpatienten bestand bzw. die Infektionsgefährdung nicht auszuschließen ist. Folgend entstehen hohe volkswirtschaftliche Kosten, aber auch die individuelle Konfrontation mit einer durchaus nicht heilbaren Infektionskrankheit.

Eine Übersichtsarbeit von Darius et al. [1] stellt eine gute Übersicht über weitere Arbeitsbelastungen des Chirurgen sowie dessen effektiven Arbeitsschutz dar. Ergänzend dazu soll diese Übersichtsarbeit auf die allgemeinen und berufsbedingten Impfempfehlungen für Chirurgen sowie auf die postexpositionelle Prophylaxe von Hepatitis B, C und HIV eingehen und vor allem neue Aspekte beleuchten.

## Eckpunkte

### Hintergrund und gesetzliche Grundlagen

Zu den häufigsten Arbeitsunfällen beim Gesundheitspersonal gehören Stich- und Schnittverletzungen^1^ [1, 2], was wiederum ein Risiko für Infektionserkrankungen darstellt [3]. Diesbezüglich zeigt sich das höchste Vorkommen der Nadelstichverletzungen bei Ärzten [3]. Bei knapp 50 % der befragten Ärzte^2^ kam mindestens eine Nadelstichverletzung innerhalb der letzten 12 Monate vor [3]. Bei Betrachtung der einzelnen Fachbereiche bot sich das höchste Vorkommen (46,9 %) von Nadelstichverletzungen bei Chirurgen. Vergleichend kamen bei Kollegen aus der HNO mit 43,5 %, der Inneren Medizin mit 40,2 % oder Anästhesie mit 32,3 % geringere Häufigkeiten von Nadelstichverletzungen vor [3]. In der Studie von Wicker et al. wurde auch die Vermeidbarkeit von Nadelstichverletzungen untersucht. Dabei war auffällig, dass ca. 45 % der Nadelstichverletzungen bei Chirurgen als nicht vermeidbar durch sichere Instrumente oder durch organisatorische Maßnahmen angegeben wurden. Vergleichend gaben Kollegen aus der HNO und der Inneren Medizin an, dass nur 3,3 % bzw. 6,5 % der Nadelstichverletzungen nicht vermeidbar gewesen wären [3].

Bedenklich ist, dass die Dunkelziffer nicht gemeldeter Nadelstichverletzungen eine hohe Spannweite zwischen 26–90 % aufweist [3–5]. Laut Weltgesundheitsorganisation erleiden jährlich ca. 3 Mio. Beschäftigte im Gesundheitswesen eine Nadelstichverletzung, von denen wiederum 16.000 eine Hepatitis-C-Virus(HCV)-, 66.000 eine Hepatitis-B-Virus(HBV)- und 1000 eine Human-immunodefiency-virus(HIV)-Infektion erleiden [3]. 90 % dieser Erkrankten leben in Entwicklungsländern [6]. Das mindert jedoch nicht die Relevanz für westliche Länder, denn die volkswirtschaftlichen Kosten für die Bundesrepublik werden auf rund 47 Mio. € geschätzt [7]. Allein die Berufsgenossenschaft für Gesundheitsdienst und Wohlfahrtspflege (BGW) verzeichnete 2019 ca. 50.000 Stich- und Schnittverletzungen [8]. Die Zahl aller meldepflichtigen und nichtmeldepflichtigen BK-3101-Fälle betrug bei der BGW im Jahr 2017 insgesamt 8612 Fälle, darunter 10 Todesfälle durch Hepatitis B (*n* = 6) und C (*n* = 2; [9]). Eine Studie an deutschen Klinikpersonal zu blutübertragenen Infektionen durch Patienten ergab für HBV eine etwa 9‑mal, für HCV eine etwa 15- mal und für HIV eine etwa 82-mal höhere Infektionsrate als für die deutsche Gesamtbevölkerung [10]. Für das Individuum selbst steht jedoch die Konfrontation mit einer durchaus nicht heilbaren Erkrankung, wie HIV, im Vordergrund.

Die Aufgabe des Arbeitgebers ist u. a. eine Gefährdungsbeurteilung laut § 5 des Arbeitsschutzgesetzes (ArbSchG 1996; [11]). Für Tätigkeiten mit biologischen Arbeitsstoffen gilt die Biostoffverordnung (BioStoffV; [12]), was auch für Gesundheitspersonal und somit auch für Chirurgen aufgrund ihrer beruflichen Arbeit am Menschen zutrifft, bei der sie mit Biostoffen^3^ in Kontakt kommen können. Außerdem hat der Arbeitgeber nach § 3 der Verordnung zur arbeitsmedizinischen Vorsorge (ArbMedVV) die Pflicht, eine angemessene arbeitsmedizinische Vorsorge zu gewährleisten und für die Beschäftigten, die mit biologischen Arbeitsstoffen umgehen, eine Pflichtvorsorge zu veranlassen [13]. Diese wird in der Regel durch einen beauftragten Arzt der Gebietsbezeichnung „Arbeitsmedizin“ oder die Zusatzbezeichnung „Betriebsmedizin“ durchgeführt. Die arbeitsmedizinischen Regel (AMR) 6,5 befasst sich zudem mit „Impfungen als Bestandteil der arbeitsmedizinischen Vorsorge bei Tätigkeiten mit biologischen Arbeitsstoffen“ [14]. Arbeitsmedizinische Indikationen für Impfungen werden aufgrund einer Gefährdungsbeurteilung gestellt [15]. Da diese Impfungen im Rahmen der arbeitsmedizinischen Vorsorge als Arbeitsschutzmaßnahmen zu betrachten sind, darf der Arbeitgeber Kosten für diese nicht dem Beschäftigten auferlegen (§ 3 Absatz 3 ArbSchG).

Ziel all dieser Maßnahmen und rechtlichen Bestimmungen ist es, Arbeitsbedingungen zu bewerten, Gefährdung zu minimieren und präventive Arbeitsschutzmaßnahmen durchzuführen. Hier ist von einer Bringschuld zu sprechen. Auf der anderen Seite ist von den Mitarbeitern zu erwarten, dass diese sich aktiv Informationen zu Arbeitsschutzmaßnahmen einholen (z. B. über Intranet, Internet, Kollegen, einschlägige Quellen der gedruckten Fachliteratur), was der sog. Holschuld entspricht. Prinzipiell wäre hier auch die Pflicht zur Meldung einer Nadelstichverletzung als Bringschuld des verletzten Arbeitnehmers anzusiedeln [7].

Für nichtimmune Beschäftigte (cave: geimpfte Nonresponder bei Hepatitis B) besteht ein Risiko für eine Hepatitis-B-Infektion von ca. 30 %, für eine Hepatitis-C-Infektion von ca. 1 % und für einer HIV-Infektion von ca. 0,3 % [4, 7, 10, 16]. Nach Infektion und chronischem Verlauf können chronisch infizierte Beschäftigte Auslöser für nosokomiale Infektionen sein. Betroffene sind grundsätzlich selbst verpflichtet, dem Arbeitgeber mitzuteilen, wenn diese eine Gefahr für Dritte darstellen können (§ 241, Absatz 2 des Bürgerlichen Gesetzbuches [BGB]; [15, 17]). Ohne eine Zustimmung des Betroffenen ist der Betriebsarzt nicht verpflichtet, eine chronische Infektionskrankheit zu melden (vgl. § 34 StGB; [18]). Er kann jedoch im Rahmen der Pflichtvorsorge den Mitarbeiter beraten und ihm empfehlen, dass er diese Informationen an den Arbeitgeber weiterleitet. Ausnahmen bestehen beim Vorliegen einer meldepflichtigen Erkrankung nach dem Infektionsschutzgesetz (IfSG; [15, 19]).

**Figure 1:**
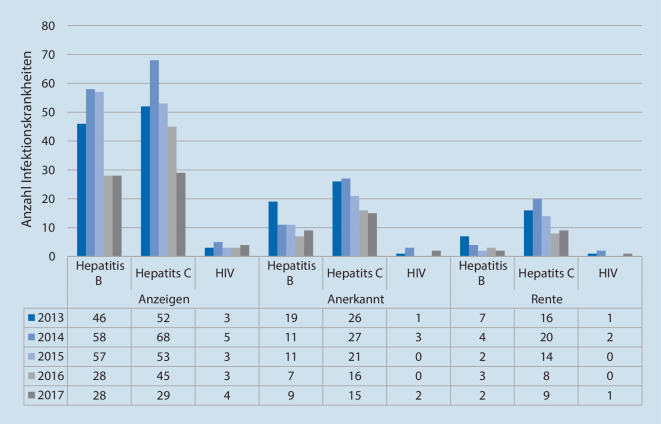

Ist die chronische Infektionskrankheit beruflich bedingt, besteht jedoch die Pflicht zur Meldung des begründeten Verdachtes auf eine berufsbedingte Infektionskrankheit nach BK 3101 an den Unfallversicherungsträger, z. B. an die Unfallkasse oder an die Berufsgenossenschaft für Gesundheitsdienst und Wohlfahrtspflege (BGW; [20]), je nachdem, wo der Beschäftigte gesetzlich unfallversichert ist. Grundlegend ist hier der § 202 des Sozialgesetzbuches (SGB) VII [21]. In Abb. 1 sind die gemeldeten Verdachtsfälle und die anerkannten Infektionskrankheiten nach BK 3101 für die Jahre 2013 bis 2017 dargestellt [22]. Es lässt sich erkennen, dass die Infektionskrankheiten Hepatitis B, C oder HIV seit 2016 leicht rückläufig sind, jedoch gehen die dokumentierten Infektionskrankheiten zum Teil mit schweren Krankheitsverläufen einher [22]. Somit sollte dieser Thematik weiterhin große Aufmerksamkeit gewidmet werden (Abb. 1).

Gerade an dieser Stelle erwähnenswert ist die beruflich bedingte Erkrankung COVID-19 durch SARS-CoV‑2. Zwar bestehen durch die Corona-Krise weniger Arbeitsunfälle, jedoch mehr Berufskrankheiten. Seit Beginn der Pandemie wurden bis Ende Februar dieses Jahres 49.295 Fälle entschieden. 42.753 Berufskrankheiten wurden anerkannt [23]. Somit stiegen die Anzeigen auf Verdacht einer Berufskrankheit (für alle BK) um rund 24 % von 41.723 auf 51.789. Abzüglich der mit Corona in Zusammenhang stehenden Erkrankungen gingen die restlichen Berufskrankheiten also leicht zurück [23].

### Allgemeine Impfempfehlungen für Erwachsene, Indikations- und Auffrischimpfungen

**Figure 2:**
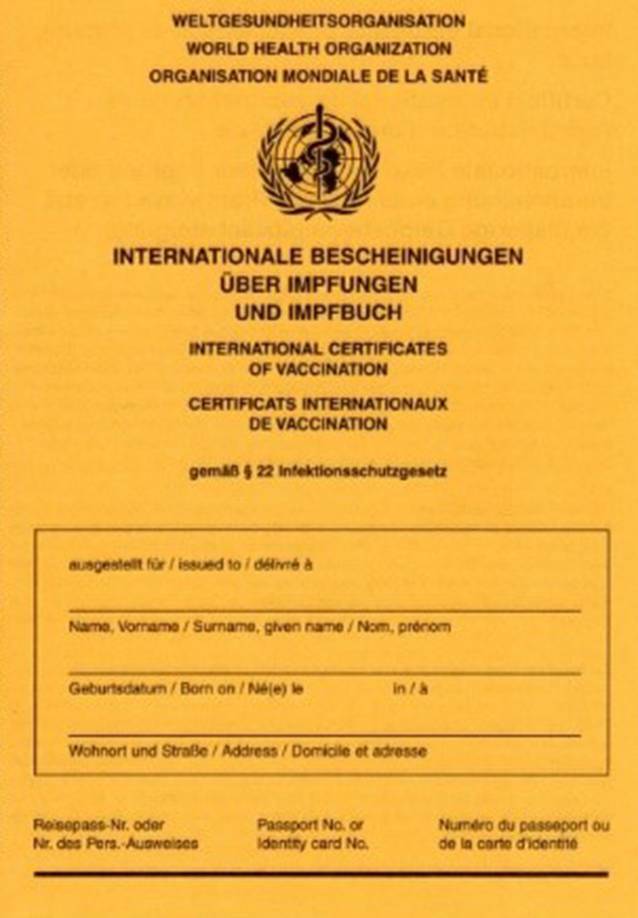

Für Kinder gibt es klare Empfehlungen, aber wann sollten sich Erwachsene gegen welche Krankheiten impfen lassen? Und welche sind besonders wichtig im Kontext der Tätigkeit im Gesundheitswesen? Diese allgemeinen Impfempfehlungen sind natürlich auch auf den Chirurgen übertragbar. Ganz klar lässt sich sagen, dass ein allumfassender Impfschutz die beste Alternative für Gesundheit ist. Idealerweise ist der Impfschutz im internationalen Impfpass (Abb. 2) dokumentiert. Die aktuelle Version des internationalen Impfpasses enthalten nun auch Dokumentationen zu Impfungen gegen COVID-19 und beruflich notwendige Impfungen. Wer seinen Impfpass nicht griffbereit hat, muss sich im ersten Schritt einen Überblick verschaffen und sollte dies bei der arbeitsmedizinischen Erstuntersuchung und im Rahmen der arbeitsmedizinischen Vorsorge besprechen. Es empfiehlt sich, den Impfpass zu jeder Untersuchung beim Betriebsarzt mitzubringen, um den Impfstatus zu kontrollieren und geimpft zu werden. Dass ist besonders hilfreich für diejenigen, die selten zum Hausarzt gehen und somit durch das klassische Hausarztmodell fallen (Abb. 2).

Die aktuellen Impfempfehlungen setzen eine bestehende Grundimmunisierung im Kindesalter voraus. Diese Standardimpfungen zum Schutz vor Tetanus (T), Diphtherie (D/d), Pertussis (aP/ap), *Haemophilus influenzae* Typ b (Hib), Poliomyelitis (IPV), Hepatitis B (HB), Pneumokokken^4^, Rotaviren (RV), Meningokokken C (MenC), Masern, Mumps, Röteln (MMR), Varizellen (V) sowie gegen humane Papillomviren (HPV) und Influenza [24]. Für einige Impfungen sind auch Frischimpfungen notwendig. Tab. 1 zeigt eine Übersicht aller Standardimpfungen und die STIKO-Empfehlung zur Auffrischimpfungen. Für schwangere Frauen können andere Voraussetzungen gelten; diese sind jedoch in der Tabelle nicht mit aufgeführt und können im *Epidemiologischen Bulletin* 34/20 nachgelesen werden [24].

**Table 1:** 

Impfung	Auffrischung	Art der Impfung	Anmerkung, Ungeimpfte oder fehlender Impfnachweis
Tetanus (T)	Alle 10 Jahre	Tdap-Kombinationsimpfung Tdap-IPV-Kombinationsimpfung	–
Diphtherie (D/d)			2 Impfungen im Abstand von 4–8 Wochen und eine 3. Impfung nach 6–12 Monaten der 2. Impfung
*Pertussis (aP/ap)*			*Personal im Gesundheitsdienst sowie in Gemeinschaftseinrichtungen*
*Poliomyelitis (IPV)*	*Alle 10 Jahre*	*IPV-Impfung* *Tdap-IPV-Kombinationsimpfung*	*Bei abgeschlossener Grundimmunisierung – wenn die letzte Impfung >* *10 Jahre zurückliegt, sollte eine einmalige Auffrischimpfung erfolgen. Für medizinisches Personal, das engen Kontakt zu Erkrankten haben kann, sollten Auffrischimpfungen alle 10 Jahre erfolgen. Ausstehende Impfungen sollen entsprechend den Angaben in den Fachinformationen mit IPV nachgeholt werden*
Haemophilus influenzae Typ b (Hib)	–	–	Personen mit anatomischer oder funktioneller Asplenie (z. B. Sichelzellanämie). Einmalige Impfung. Unzureichende Datenlage zu Wiederholungsimpfungen
*Hepatitis B (HB)*	*Wenn Titer <* *100* *IE/l*	*–*	*4–8 Wochen nach der 3. Impfung Anti-HBs quantitativ bestimmt: erfolgreiche Impfung: Anti-HBs ≥100* *IE/l, „Low-Responder“ (Anti-HBs 10–99* *IE/l) sofortige weitere Impfstoffdosis mit erneuter Anti-HBs-Kontrolle nach weiteren 4–8 Wochen, weitere 2 Impfdosen mit anschließender Anti-HBs-Kontrolle nach 4–8 Wochen. „Nonresponder“ (Anti-HBs <10* *IE/l) -Bestimmung von HBsAg und Anti-HBc zum Ausschluss einer bestehenden chronischen HBV-Infektion. Wenn negativ, weiter wie „Low-Responder“*
*Hepatitis A (HA)*	*–*	*Monovalente Impfstoffe und Kombinationsimpfstoffe mit Typhus oder Hepatitis B*	*Gesundheitsdienst (inkl. Sanitäts- und Rettungsdienst). Grundimmunisierung nach Fachinformation. 2 Impfdosen im Abstand von 6–12 Monaten. Auffrischung noch nicht völlig geklärt, ob sinnvoll*
Herpes zoster (HZ)	Personen ≥ 60 Jahre	Adjuvantierter Totimpfstoff	2‑mal Impfung im Abstand von mindestens 2 bis maximal 6 Monaten. Personen ≥ 50 Jahre bei erhöhter gesundheitlicher Gefährdung infolge einer Grundkrankheit
Pneumokokken	Personen ≥ 60 Jahre	23-valenter Polysaccharidimpfstoff (PPSV23)	Ggf. Wiederholung nach 6 Jahren
	Erhöhte gesundheitliche Gefährdung	13-valenter Konjugatimpfstoff (PCV13) + PPSV23	1. Impfung mit 13-valenten Konjugatimpfstoff (PCV13), gefolgt von PPSV23 nach 6–12 Monaten
Meningokokken C (MenC)	–	–	Keine routinemäßige Empfehlung. Gesundheitlich gefährdete Personen mit angeborener oder erworbener Immundefizienz bzw. -suppression; es ist fraglich, ob diese als Chirurgen arbeiten
*Masern*	*–*	*MMR-Impfung*	*2‑mal Impfung als Grundimmunisierung. Nach 1970 geborenes Personal mit unklarem Impfstatus, ohne Impfung oder mit unvollständiger Impfung: 2 Impfdosen ggf. mit Varizellenimpfung MMR(V)-Impfstoff. Impfpflicht seit 01.03.2020 [*34*] für Personal in medizinischen Einrichtungen (gemäß § 23 (3) Satz 1 IfSG) inklusive Einrichtungen sonstiger humanmedizinischer Heilberufe*
*Mumps*	*–*		
*Röteln*	*–*		
*Varizellen (V)*	*–*	*MMR(V)-Impfung*	*2‑mal bei seronegativen Personen, beruflich bedingt in medizinischen Einrichtungen bzw. Kontakt zu potenziell infektiösem Material*
*Influenza*	Personen ≥ 60 Jahre	Inaktivierter quadrivalenter Hochdosisimpfstoff	Jährliche Impfung im Herbst
	*Personen mit erhöhter Gefährdung, z.* *B. medizinisches Personal, erhöhte gesundheitliche Gefährdung*	*Inaktivierter quadrivalenter Impfstoff*	*Jährliche Impfung im Herbst*

*Kursiv* Impfungen, die besonders für Chirurgen aufgrund ihrer beruflichen Tätigkeit regelmäßig aufgefrischt werden sollten

### Impfungen aufgrund eines erhöhten beruflichen Risikos am Beispiel des Chirurgen

Beruflich indizierte Impfungen gehören der Kategorie B an, also aufgrund eines erhöhten Risikos im Zusammenhang mit der Tätigkeit. Dieses wird nach Empfehlungen nach erfolgter Gefährdungsbeurteilung gemäß ArbSchG [11] sowie Berücksichtigung der BioStoffV [12] und ArbMedVV [13] und/oder zum Schutz Dritter im Rahmen der beruflichen Tätigkeit durchgeführt [24]. Dabei zeigen die kursiv ausgezeichneten Zellen der Tab. 1 Impfungen, die besonders für Chirurgen aufgrund ihrer beruflichen Tätigkeit regelmäßig aufgefrischt werden sollten (Tab. 1).

### Primärprophylaxe, Impfempfehlungen und Postexpositionsprophylaxe nach stattgehabten Stich- oder Schnittverletzungen

Zu den Aufgaben des Betriebsarztes gehört es auch, den Arbeitgeber zur Organisation und Qualitätssicherung der Nachsorge eines Arbeitsunfalles – hier im Sinne einer Nadelstichverletzung – zu beraten [15]. Im Akutfall ist dieser jedoch nicht der erste Ansprechpartner. Die Akutversorgung einer Nadelstichverletzung ist ein Notfall [25] und erfolgt durch einen D‑Arzt, jedoch kann im Vorfeld, gemeinsam mit den zuständigen D‑Ärzten und ggf. dem Labor, die grundsätzliche Verfahrensweise für ein Schadensereignis festgelegt werden [15]. Außerdem kann der Betriebsarzt Informationen, die für die Unterweisung der Beschäftigten im Rahmen notwendiger Handlungsschritte bei Nadelstichverletzungen notwendig sind, aufbereiten [15].

Die Akutversorgung einer Nadelstichverletzung umfasst auch eine Risikoanalyse mit Untersuchung der Indexperson. Diese Untersuchung ist jedoch nicht Voraussetzung zur Nachsorge der verletzten Person; eine Aufklärung und Einverständnis werden empfohlen [26]. Sofort nach dem Übertragungsereignis sollten folgende Serologien aus dem Blut der Indexperson bestimmt werden:

1. HBsAg und Anti-HBc (Anti-HBs) nur, wenn bei der verletzter Person kein sicherer HBV-Immunschutz besteht,
2. Anti-HCV oder HCV-NAT bei positiver oder bei unzureichender antiviraler Behandlung der Indexperson sowie bei immundefizienter Indexperson (und)
3. HIV-Screeningtest der 4. Generation oder Bestimmung der Viruslast mittels HIV-NAT bei positivem Indexpatienten [27].

Die *Hepatitis-B-Virus-*Übertragung im beruflichen Kontext des Chirurgen erfolgt parenteral über Blut, Blutprodukte, Sekrete und Exsudate. Bei einer Nadelstich- oder Schnittverletzung besteht hohes Übertragungsrisiko; durchaus bis zu 100 % bei einem positiven Envelope-Antigen-Träger (HBe-Ag; [5]). Die Inkubationszeit liegt bei 30 bis 180 Tagen je nach Viruslast [28]. Für chirurgisch tätige Ärzte ist ein vollständiger Impfschutz gegenüber Hepatitis B zu empfehlen. Das heißt mindestens 3‑malige Impfung mit Nachweis von Anti-HBs > 100 IU/I in den letzten 10 Jahren, was eine gesicherte Immunität bedeutet. Für diese Beschäftigten ergeben sich keine Akutmaßnahmen nach Nadelstichverletzung. Bei unsicherer Immunität werden die passive und aktive Immunisierung nach STIKO empfohlen [20, 27]: simultan Hepatitis-B-Immunglobulin intragluteal und Hepatitis-B-Impfstoff intradeltoidal. Die Schutzimpfung wird während der nächsten Monate gemäß den Vorschriften des Impfstoffherstellers für die Grundimmunisierung komplettiert [29]. Für den Fall, dass sich der Beschäftigte an einer unbekannten, gebrauchten Kanüle gestochen oder sonstiger Blutkontakt bestand und eine zweifelhafte Immunität besteht, sollte ein Schnelltest mit Bestimmung des Anti-HBs-Status erfolgen und bei fehlender Immunität ebenfalls simultan geimpft werden [29]. Nonresponder erhalten bei Exposition unverzüglich simultan HB-Impfstoff und HB-Immunglobulin [29].

Die Übertragung des *Hepatitis-C-Virus* im Setting chirurgischer Arbeitsplatz ist ähnlich wie bei Hepatitis B. Das Risiko einer Übertragung bei beruflichem Kontakt liegt bei etwa 2–10 % und ist damit niedriger als das Risiko bei einer Hepatitis B. Die Inkubationszeit beträgt zwischen 15 Tagen und 4 bis 6 Monaten [28]. Weder eine Impfung noch eine Postexpositionsprophylaxe (PEP) steht für die Hepatitis-C-Infektion [5] zur Verfügung. Ein HCV-Nukleinsäure-Amplifikationstest (HCV-NAT) bietet nach 4 bis 6 Wochen eine große diagnostische Sicherheit [27] und wird bei erhöhtem Risiko, HCV-infektiöser oder unbekannter Indexperson durchgeführt [27]. Ansonsten erfolgt die Bestimmung von Anti-HCV [27].

Auch bei der *HIV-Infektion* erfolgt die Übertragung parenteral durch die Inokulation von infektiösem Blut, Blutprodukten oder anderen Körperflüssigkeiten sowie über die Konjunktiven. Die Inkubationszeit beträgt 2 bis 6 Wochen nach Infektion und nach etwa 2 Wochen sind Antikörper gegen HIV nachweisbar [20]. Auch für HIV existieren keine Impfungen, jedoch ist eine PEP möglich. Nach Kontakt zu einem HIV-positiven Indexpatienten sollte zügig eine HIV-PEP begonnen werden, optimalerweise sollte sie innerhalb der ersten 2 h nach Nadelstichverletzung erfolgen [5, 27]. HIV-AK-Testungen der 4. Generation sind sofort, nach 6 und 12 Wochen durchzuführen und eine Triple-Therapie für 4 Wochen zu initiieren, die unmittelbar nach Exposition begonnen werden sollte [28]. Zwei negative HIV-Tests schließen eine HIV-Infektion nach 12 bzw. 16 Wochen mit großer Sicherheit aus ([27], Tab. 2).

**Table 2:** 

	HBV	HCV	HIV
Sofort	Anti-HBc, Anti-HBs bei unsicherer Immunität	Anti-HCV	HIV-Screeningtest 4. Generation
PEP	Impfstoff + Immunglobuline	–	Triple-Therapie für 4 Wochen
Nach 6 Wochen	Anti-HBs nach Impfung, Titer > 100 IE/l keine weiteren Tests Bei unsicherer Immunität: HbsAg, Anti-HBc	Anti-HCV, HCV-NAT	HIV-Screeningtest 4. Generation, bei HIV-PEP erst nach 10 Wochen
Nach 12 Wochen	Bei unsicherer Immunität: Anti-HBc und Anti-HBs	Anti-HCV	HIV-Screeningtest 4. Generation, bei HIV-PEP erst nach 16 Wochen
Nach 6 Monaten			Entfällt nach 2 negativen HIV-Screeningtests der 4. Generation in der 6./12. Woche oder 10./16. Woche nach 4‑wöchiger HIV-PEP

*HBV* Hepatitis-B-Virus, *HCV* Hepatitis-C-Virus, *HIV* Humane Immundefizienz-Virus, *Ag* Antigen

An dieser Stelle soll die Empfehlung für eine COVID-19-Impfung nicht unerwähnt bleiben. Chirurgen gehören zu „Personen, die in Bereichen medizinischer Einrichtungen oder im Rahmen der Ausübung eines Heilberufes mit einem hohen oder erhöhten Expositionsrisiko in Bezug auf das Coronavirus SARS-CoV‑2 tätig sind, insbesondere Ärztinnen und Ärzte und Personal mit regelmäßigem Patientenkontakt … [30]“, somit besteht eine berufliche Indikation.

*Primäre Schutzmaßnahmen *orientieren sich am TOP-Prinzip, welches technische, organisatorische und personenbezogene Schutzmaßnahmen umfasst [31] und hier auf die Vermeidung von Nadelstichverletzungen übertragbar ist.

Zu den *technischen* Maßnahmen gehören das Verwenden stichsicherer Nadelsysteme und Instrumente [5]. Stichsichere Instrumente bieten folgende Merkmale [32]:

- Sicherheitsmechanismus ist Bestandteil des Instrumentes,
- Aktivierung muss leicht erkennbar und hörbar sein sowie schnell und mit einer Hand durchführbar sein.

Außerdem sollte der Sicherheitsmechanismus nicht reversibel sein (kein „recapping“). Dazu gehört auch die Nutzung von Einwegmaterial. Gerade bei infektiösen Patienten ist das Tragen doppelter Handschuhe zu empfehlen [33].

Zu den *organisatorischen* Maßnahmen gehören die:

- Beachtung und Unterweisung in die Sicherheitshinweise der Instrumente,
- Vermeidung von Zeitdruck (und)
- sachgerechte Instrumentenübergabe am Operationstisch (sowie)
- sachgerechte Handhabung der Abwurfbehälter [31].

Zu den *personenbezogenen *Schutzmaßnahmen gehören neben der medikamentenösen Therapie (z. B. Schutzimpfung) auch folgende Maßnahmen, die unverzüglich und ohne Verzug einzuleiten sind [2]:

- Erhöhung des Blutflusses durch Druck auf das umliegende Gewebe,
- antiseptische Spülung und Nachspülung und Anlegen eines antiseptischen Wirkstoffdepots,
- Eruierung und Untersuchung des Indexpatienten,
- Aufsuchen eines D‑Arztes und Einleitung eines D‑Arzt-Verfahrens (prinzipiell Notfall [25]) (sowie)
- zeitnahe Vorstellung beim Betriebsarzt [2].

## Fazit

- Ein voller Impfschutz ist die beste Grundlage für die Gesundheit(erhaltung), denn Chirurgen gehen einer beruflichen Tätigkeit nach, die infektionsgefährdend ist.
- Neben den allgemeinen Impfempfehlungen besteht auch eine Reihe beruflich bedingter Impfungen, die im Rahmen der arbeitsmedizinischen Pflichtvorsorge kontrolliert und ggf. durchgeführt werden können.
- Nadelstichverletzung sollten nicht verharmlost werden und bei kontaminierten Instrumenten sollte zügig ein D‑Arzt aufgesucht werden, um ggf. eine postexpositionelle Prophylaxe einzuleiten und damit etablierte Therapieansätze nutzbringend auszuschöpfen sowie auch mittelfristigen und Spätkonsequenzen vorzubeugen.

## Footnotes

1 Zur besseren Lesbarkeit wird hier nur von Nadelstichverletzungen gesprochen. Sofern nicht anders angegeben, bezieht sich der Text auf Nadel- und Schnittverletzungen durch kontaminierte Instrumente.

2 Aus Gründen der besseren Lesbarkeit wird auf die gleichzeitige Verwendung männlicher und weiblicher Sprachformen verzichtet. Sämtliche Personenbezeichnungen gelten gleichermaßen für alle Geschlechter, wenn nicht explizit auf männliche oder weibliche Personen verwiesen wird.

3 Biostoffe nach BioStoffV sind u. a. auch Mikroorganismen, Zellkulturen und Endoparasiten einschließlich ihrer gentechnisch veränderten Formen.

4 Impfempfehlung vor dem 61. Lebensjahr bei erhöhter gesundheitlicher Gefährdung infolge einer Grundkrankheit.
